# Prevalence of *Propionibacterium acnes* in Intervertebral Discs of Patients Undergoing Lumbar Microdiscectomy: A Prospective Cross-Sectional Study

**DOI:** 10.1371/journal.pone.0161676

**Published:** 2016-08-18

**Authors:** Manu N. Capoor, Filip Ruzicka, Tana Machackova, Radim Jancalek, Martin Smrcka, Jonathan E. Schmitz, Marketa Hermanova, Jiri Sana, Elleni Michu, John C. Baird, Fahad S. Ahmed, Karel Maca, Radim Lipina, Todd F. Alamin, Michael F. Coscia, Jerry L. Stonemetz, Timothy Witham, Garth D. Ehrlich, Ziya L. Gokaslan, Konstantinos Mavrommatis, Christof Birkenmaier, Vincent A. Fischetti, Ondrej Slaby

**Affiliations:** 1 Laboratory of Bacterial Pathogenesis and Immunology, Rockefeller University, New York, NY, United States of America; 2 Department of Microbiology, Faculty of Medicine, Masaryk university, St. Anne’s Faculty Hospital, Brno, Czech Republic; 3 Department of Molecular Oncology, Central European Institute of Technology (CEITEC), Masaryk University, Brno, Czech Republic; 4 Department of Neurosurgery, St. Anne’s University Hospital, Masaryk University, Brno, Czech Republic; 5 Department of Neurosurgery, University Hospital Brno, Masaryk University, Brno, Czech Republic; 6 Department of Pathology, Microbiology and Immunology, Vanderbilt University School of Medicine, Nashville, TN, United States of America; 7 1st Department of Pathological Anatomy, St. Anne’s University Hospital, Masaryk University, Brno, Czech Republic; 8 Department of Neurosurgery, University Hospital Ostrava, Ostrava University, Ostrava, Czech Republic; 9 Department of Orthopedic Surgery, Stanford University Medical Center, Stanford University, Stanford, CA, United States of America; 10 Department of Orthopedic Surgery, OrthoIndy Hospital, Indianapolis, IN, United States of America; 11 Department of Anesthesia, The Johns Hopkins Hospital, Baltimore, MD, United States of America; 12 Department of Neurosurgery, The Johns Hopkins Hospital, Baltimore, MD, United States of America; 13 Center for Genomic Sciences, Drexel University College of Medicine, Philadelphia, Pennsylvania, United States of America; 14 Department of Microbiology and Immunology, Drexel University College of Medicine, Philadelphia, Pennsylvania, United States of America; 15 Department of Otolaryngology Head and Neck Surgery, Drexel University College of Medicine, Philadelphia, Pennsylvania, United States of America, and Institute of Molecular Medicine and Infectious Disease, Drexel University College of Medicine, Philadelphia, PA, United States of America; 16 Department of Neurosurgery, The Warren Alpert Medical School of Brown University, Rhode Island Hospital, Providence, RI, United States of America; 17 Celgene Corporation, Information Knowledge and Utilization, San Francisco, CA, United States of America; 18 Department of Orthopedics, Physical Medicine & Rehabilitation, University of Munich (LMU), Munich, Germany; Aarhus Universitet, DENMARK

## Abstract

**Background:**

The relationship between intervertebral disc degeneration and chronic infection by *Propionibacterium acnes* is controversial with contradictory evidence available in the literature. Previous studies investigating these relationships were under-powered and fraught with methodical differences; moreover, they have not taken into consideration *P*. *acnes’* ability to form biofilms or attempted to quantitate the bioburden with regard to determining bacterial counts/genome equivalents as criteria to differentiate true infection from contamination. The aim of this prospective cross-sectional study was to determine the prevalence of *P*. *acnes* in patients undergoing lumbar disc microdiscectomy.

**Methods and Findings:**

The sample consisted of 290 adult patients undergoing lumbar microdiscectomy for symptomatic lumbar disc herniation. An intraoperative biopsy and pre-operative clinical data were taken in all cases. One biopsy fragment was homogenized and used for quantitative anaerobic culture and a second was frozen and used for real-time PCR-based quantification of *P*. *acnes* genomes. *P*. *acnes* was identified in 115 cases (40%), coagulase-negative *staphylococci* in 31 cases (11%) and alpha-hemolytic streptococci in 8 cases (3%). *P*. *acnes* counts ranged from 100 to 9000 CFU/ml with a median of 400 CFU/ml. The prevalence of intervertebral discs with abundant *P*. *acnes* (≥ 1x10^3^ CFU/ml) was 11% (39 cases). There was significant correlation between the bacterial counts obtained by culture and the number of *P*. *acnes* genomes detected by real-time PCR (r = 0.4363, p<0.0001).

**Conclusions:**

In a large series of patients, the prevalence of discs with abundant *P*. *acnes* was 11%. We believe, disc tissue homogenization releases *P*. *acnes* from the biofilm so that they can then potentially be cultured, reducing the rate of false-negative cultures. Further, quantification study revealing significant bioburden based on both culture and real-time PCR minimize the likelihood that observed findings are due to contamination and supports the hypothesis *P*. *acnes* acts as a pathogen in these cases of degenerative disc disease.

## Introduction

*Propionibacterium acnes (P*. *acnes)* is a facultative anaerobic, Gram-positive, slow-growing, fastidious bacteria [1,2] prevalent on human skin and sebaceous ducts as part of the normal flora [3,4,5] and is implicated in the development of acne vulgaris [6] as well as postoperative and device-related infections [7]. *P*. *acnes* have also been identified in the oral cavity, gastrointestinal tract, genitourinary tract, eye (conjunctiva), and external ear canal [1]. *P*. *acnes* may play a role in other conditions, including prostatitis [8], SAPHO [9], sarcoidosis [10], and degenerative disc disease [11].

The pioneering study published in 2001 by Stirling *et al*. in Lancet, reported that 53% of the patients with severe sciatica had Gram-positive anaerobic microorganisms in their disc tissue of which 84% were *P*. *acnes* [12]. Considering the potential clinical consequences of a *P*. *acnes* infection as an etiological factor in degenerative disc disease, these findings are indicative of potentially significant similarities to the discovery of *Helicobacter pylori* involvement in pathogenesis of peptic ulcers and the shift it led to in the way peptic ulcers are treated [13]. Subsequently, several independent studies have demonstrated the presence of infected extruded disc tissue from first-time disc herniations, with *P*. *acnes* being the most commonly involved organism [14–18]. According to a meta-analysis of these studies from 2015, *P*. *acnes* was the most prevalent bacteria in disc tissue, with the pooled estimate of the proportion with positive samples being 34% [19]. More recently, the first experimental evidence was provided showing that *P*. *acnes* has the ability to induce disc degeneration in a rabbit model [20]. Other studies, however, reported very low or zero prevalence of bacterial infection in samples from patients with lumbar disc herniation, quoting contamination as the predominant source [21–23].

Contributing to this lack of consensus, significant methodical differences exist among studies. The most notable of which is the lack of laboratory procedures that take into account a possible biofilm mode of growth associated with *P*. *acnes* infection. This translates into inconsistencies in regard to tissue homogenization procedures to break up biofilm aggregates, leading to negative culture results. As a consequence, *P*. *acnes* positivity was likely underestimated in some of the previous studies [21, 23]. None of the studies implemented quantitative culture or real-time PCR-based quantification of *P*. *acnes* genomes. We believe that quantitation of the bacterial bioburden is an important criterion that can contribute to discrimination between truly infected and skin-contaminated cases. Thus, a lack of quantitative methods to identify *P*. *acnes* could have led to overestimating the positivity rates in previous studies. These methodical weaknesses together with the small sample sizes (only one study enrolled more than 100 patients for bacterial culture [23]) have left these studies underpowered, with limited applicability of their results towards clinical decision-making or any definitive conclusions.

In this prospective cross-sectional study on the currently largest series of patients undergoing microdiscectomy for lumbar disc herniation, we implemented disc tissue homogenization, quantitative microbiologic culture, real-time PCR-based detection of *P*. *acnes* genomes, and procedural DNA contamination controls to determine *P*. *acnes* counts in disc tissue samples and evaluated the prevalence of intervertebral discs with an abundance of *P*. *acnes*.

## Methods

### Study design and patients

Between May 2015 and December 2015, adults scheduled for microdiscectomy for symptomatic lumbar disc herniation at University Hospital Brno (Brno, Czech Republic) and St. Anne’s University Hospital (Brno, Czech Republic) were prospectively screened for potential participation in this cross-sectional study. Inclusion criteria included: lumbar or lumbosacral radiculopathy with or without sensory deficits but with either a matching, clinically relevant motor deficit in correlating lumbar or sacral nerve root distributions (see imaging criteria) or with radicular pain (sciatica or femoralgia) that was intractable by conservative means; matching physical examination findings including positive straight leg raise test, dermatomal sensory deficits, myotomal motor deficits and/or a diminished deep tendon reflexes; current magnetic resonance imaging or computed tomography imaging of the lumbosacral spine showing a free nucleus pulposus sequestration or a disc herniation / protrusion in a distribution correlating with the clinically affected nerve roots and with the physical examination. Exclusion criteria included: coexistent infection or immunologically compromised conditions; corticosteroid or antibiotics use in the month before surgery; trauma; unknown radiographic mass; diagnosis of inflammatory arthritis or other rheumatologic diseases. The following epidemiological and clinical data were collected: Gender, age, intervertebral segment involved, type of herniation, prevalence of previous spinal surgeries, prior epidural steroid injections, and development of post-operative discitis. A written informed consent was obtained from each patient. The study was approved by the Institutional Review Boards of University Hospital Brno and St. Anne’s University Hospital Brno.

### Collection of intraoperative samples

The surgical site was scrubbed with triple preparation of povidone iodine and draped using standard sterile technique. Standard perioperative antibiotics were given before the skin incision in all cases. Cefazolin was the standard antibiotic given in most cases. In penicillin-allergic patients, either vancomycin or clindamycin was administered. The precise location for the skin incision was guided by intraoperative fluoroscopy and a posterior midline approach using sharp dissection and electrocautery was performed. After placement of a self-retaining retractor (Caspar type) and under an operating microscope, ligamentum flavum was resected as required by means of Penfield dissectors and Kerrison rongeurs. The disc herniation was exposed by gentle retraction of the traversing nerve root and then removed together with the remnant loose fragments of the nucleus pulposus in the disc space near the annular defect. All tissue samples were handled in such a way as to minimize their contamination, retained in a closed sterile sample cup, and then passed off to the field for labeling and transport to the Department of Microbiology at St. Anne’s University hospital (Brno, Czech Republic), where the disc tissues samples from both centers were further analyzed. The sample sizes were approximately 3x3x5-10x5x5 mm and not measured accurately as we attempted to obtain as much material as the possible from the surgical specimen. Samples were not frozen prior to processing and culture was established within 2 to 4 hours post-surgery.

### Microbiological culture

Fresh disc tissue samples were cut into smaller fragments using a sterile, individually packaged, gamma-irradiated scalpel; and, a sterile, gamma-irradiated petri dish. One of these fragments was placed into a 2 mL microcentrifuge DNA-free tube and stored at -80°C until processed for *P*. *acnes* DNA analysis. Tissue processing and homogenization was carried out in a sterile pestle and mortar with sterile quartz sand (size particle 0.1–0.5 mm; Penta, Czech Republic) and saline solution in aseptic conditions, in a class 2 biological safety cabinet. The homogenized tissue samples were inoculated onto Wilkins Chalgren Anaerobic Agar (Hi Media Laboratories, India) with 7% of sheep’s blood and vitamin K. Inoculated plates were incubated anaerobically (80% nitrogen, 10% CO_2_ and 10% H_2_) in an Anaerobic Work Station (Ruskinn Technology, UK) at 37°C for 14 days and assessed for bacterial growth. The quantity of bacteria in the sample was expressed as colony forming units (CFU) in 1 ml of the homogenate. Identification of bacteria was carried out biochemically using the Rapid ANA II System (Remel, USA).

### Quantitative real-time PCR

Frozen tissue samples were thawed (median wet weight was 130 mg, range 20–180 mg), cut into small fragments and transferred to a sterile 2 mL microcentrifuge tube by use of newly opened sterile sets of needle, scalpel and tweezers. Small fragments of the tissue samples were further suspended in 500 μl of ATL buffer (Qiagen, Germany) with 50 μl of proteinase K (20 mg/ml) (Qiagen) and digested at 56°C and 650 rpm in a thermomixer overnight. To each set of the samples that were processed in parallel, we used a tube with sterile water as a laboratory contamination control to follow the entire laboratory process from digestion of the tissue and DNA isolation to real-time PCR analysis. DNA was extracted by use of the QIAamp UCP Pathogen Mini Kit (Qiagen) as described in the manufacturer’s instructions. Concentration of DNA were measured spectrophotometrically using a Nanodrop 2000 (Thermofischer, USA); or with fluorescent dye and a Qubit 3.0 fluorometer (Life Technologies, USA) for samples with DNA concentrations less than 5ng/μl. A previously described real-time PCR assay was performed using primers to amplify a 131-bp region of the 16S rRNA gene of *P*. *acnes*: forward primer 5’- GCGTGAGTGACGGTAATGGGTA -3’, reverse primer 5’-TTCCGACGCGATCAACCA-3’ and TaqMan probe 5’-AGCGTTGTCCGGATTTATTGGGCG-3’ [24]. The 15-μl PCR reaction mixture contained 6,75 μl of DNA sample, 5 pmol of each primer and 2 pmol of TaqMan probe, and 1X TaqMan Gene Expression Master Mix (Life Technologies, USA). The QuantStudio 12K Flex system (Life Technologies, USA) was used with the thermal cycling profile of 50°C for 2 min, 95°C for 10 min and 50 cycles of 95°C for 15 s and 60°C for 1 min. The *P*. *acnes* genome equivalents in samples were estimated with an internal standard curve prepared with five replicates of six concentrations (10–10^6^ copies) of synthesized *P*. *acnes* amplicon (131 bp) (Integrated DNA Technologies, USA). Laboratory contamination controls described above and PCR negative controls were included in every PCR reaction. Assays were done in duplicate for each sample, and the mean number of the 16S rRNA gene copies was calculated. To eliminate laboratory contamination, 16S rRNA counts detected in laboratory contamination control were subtracted from the copies number in the tissue samples. The number of bacterial genomes in each sample was finally calculated using the known number of copies of the 16S rRNA operon (3 copies/cell) in *P*. *acnes* [4] and represented as the number of bacterial genomes in 500 ng of total DNA extracted from the disc tissue sample. Human ß-globin gene was included as an internal control to allow assessment of the specimen quality and the nucleic acid extraction as well as the inhibition amplification process as described previously [24].

### Data analysis

A description of identified bacterial frequencies was performed and the relationship between presence/counts of *P*. *acnes* and clinical parameters was evaluated. Categorical variables were analyzed using a two-sided Fisher’s exact test, continuous variables (genome counts, age) with the non-parametric Mann-Whitney U test. Spearman’s correlation was used to test the association between bacterial counts reached in the same samples by different methods. The significance level was set at 0.05. The statistical software used was Prism 5 (GraphPad Software Inc., USA).

## Results

### Patient characteristics

We prospectively enrolled 290 patients (171 males and 119 females) with an average age of 47 ± 13 years. One hundred ninety-two cases were enrolled at University Hospital Brno, ninety eight cases at St. Anne’s University Hospital. The degenerative intervertebral disc segments were L2/L3 in 8 (3%), L3/L4 in 21 (7%), L4/L5 in 137 (47%) and L5/S1 in 124 (43%) cases. For 8 patients who underwent a double-level disk surgery, only the caudal disk was selected to avoid a statistical bias. Disc herniation types included disc protrusion in 23 (8%), disc extrusion in 126 (44%), and disc sequestration in 141 (48%) patients. Medical history of previous spinal surgery was noted in 51 (17%) cases, prior epidural steroid injection was received by 14 (5%) patients. None of the patients developed clinically evident post-operative discitis. There were no significant differences in patient characteristics between the two recruitment centers regarding age, gender and disease-specific clinical data which were collected and used in our study.

### Results of bacterial culture

**Fig 1:**
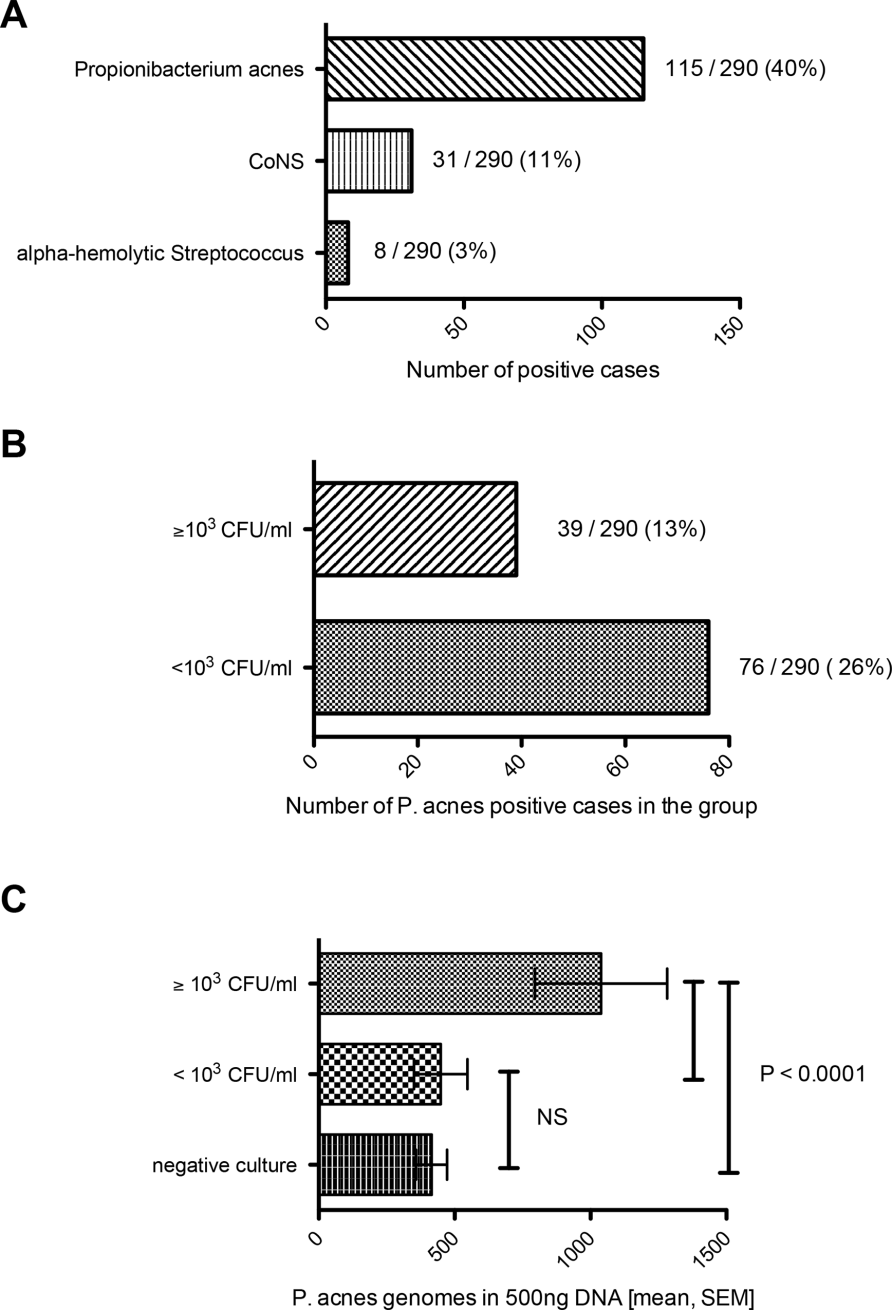
Results of anaerobic culture and detection of *P*. *acnes* genome counts in disc tissue samples. Prevalence rate of the bacteria isolated from disc tissue samples (A), stratification of the samples accordingly to bacterial counts reached by culture (B) and differences in the bacteria genome counts detected by real-time PCR in group of patients with abundant *P*. *acnes* by culture, non-abundant *P*. *acnes* and culture-negative cases (C).

Bacteria were identified in 130 of 290 disc tissues samples, the other 160 biopsies resulted in no bacterial colonies. The following bacteria were isolated: *P*. *acnes* in 115 cases (40%), coagulase-negative staphylococci (*CoNS*) in 31 cases (11%) and *alpha-hemolytic streptococci* (*AHS*) in 8 cases (3%) (Fig 1A). Twenty-four patients (8%) had two microorganisms present in the disc. No patients had more than two types of bacteria isolated. We observed significant variability in *P*. *acnes* counts among positive disc samples ranging from 100 to 9000 CFU/ml (median 400 CFU/ml).

A threshold was defined as ≥ 1x10^3^ CFU/ml (75^th^ percentile) for the discs with abundant *P*. *acnes*. Prevalence of the disc samples with abundant *P*. *acnes* was 11% (39 cases); in the remaining 29% (76 cases) of positive disc samples *P*. *acnes* was not abundant (< 1x10^3^ CFU/ml).

### *Propionibacterium acnes* genome counts in disc tissue samples

DNA was successfully extracted from all samples included in the study with concentration ranging from 2 to 45 ng/μl (median 13 ng/μl). The mean Ct (threshold cycle) value for the internal control, human ß-globin gene, was 24.03 ± 1.81. Eighteen cases of the 290 cases were excluded from statistical evaluation due to negative of ß-globin gene (Ct > 35). The mean *P*. *acnes* genomes count in the laboratory contamination control samples was 4 ± 4 genomes. *P*. *acnes* genomes were undetectable in only 13 (4%) cases. The number of *P*. *acnes* genomes in the 259 *P*. *acnes*-positive disc tissues ranged from 2 to 5831 with a median of 260 genomes per 500 ng of total DNA. There were significant differences in the number of *P*. *acnes* genomes detected in the samples with abundant *P*. *acnes* by culture (1039 ± 244 genomes), when compared to samples with non-abundant *P*. *acnes* (448 ± 98 genomes) and samples negative by culture (415 ± 57 genomes) (for both P < 0.0001, Fig 1C). We also found significant Pearson correlation between CFU/ml and *P*. *acnes* genome counts detected by real-time PCR in patients positive by culture (r = 0.4363, p<0.0001). There was no difference in the number of *P*. *acnes* genomes between disc tissue samples with non-abundant *P*. *acnes* and samples negative by culture (p = 0.6973).

### Relationships of *Propionibacterium acnes* and patient characteristics

The average age of patients having discs with abundant *P*. *acnes* by culture was significantly lower (42 versus 48 years; p = 0.0086). There were no significant differences when comparing the groups of patients (*i*.*e*., *abundant P*. *acnes*, *non-abundant P*. *acnes*, *or negative cultures*) in gender, prevalence of previous spinal surgery, prevalence of prior epidural steroid injection, distribution of herniation type, or affected intervertebral level (summarized in Table 1). With the exception of the age, there were no significant associations found between *P*. *acnes* prevalence and clinical characteristics including the situation in which all patients with a positive culture (abundant + non-abundant) were considered positive for statistical evaluation or the *P*. *acnes* genome counts were compared between groups (all p > 0.05).

**Table 1:** Relationships between strong positivity of *P*. *acnes* in the disc and epidemiological/clinical characteristics of the patients.

Parameters	Abundant *P*. *acnes* by culture N (%)	Non-abundant *P*. *acnes* or negative culture N (%)	P-value
**Gender**			
Male	28 (16%)	144 (84%)	0.1144
Female	11 (9%)	108 (91%)	
**Age**			
Mean ± SD	42 ± 12 y	48 ± 13 y	**0.0086**
**Previous spinal surgery**			
Yes	8 (16%)	43 (84%)	0.5019
No	30 (14%)	209 (86%)	
**Prior epidural injection**			
Yes	3 (21%)	11 (79%)	0.4128
No	111 (40%)	165 (60%)	
**Type of herniation**			
Protrusion	3 (13%)	20 (87%)	0.6445
Extrusion	14 (11%)	112 (89%)	
Sequestration	21 (15%)	119 (85%)	
**Intervertebral level**			
L2/L3	1 (13%)	7 (87%)	0.6313
L3/L4	1 (5%)	20 (95%)	
L4/L5	15 (16%)	81 (84%)	
L5/S1	23 (14%)	142 (86%)	

*CoNS*, coagulase-negative *staphylococci*.

## Discussion

Traditionally, mechanical, degenerative (age-related), traumatic and genetics-based theories have been proposed as possible etiologies of degenerative disc disease [25], but evidence has emerged over the last decade showing that low-grade infections, mostly by *P*. *acnes*, playing a potential causative role in disc degeneration and resultant disc-herniation and radicular pain [11, 19]. However, the potential relationship between intervertebral disc degeneration and chronic infection by low-virulence *P*. *acnes* remains controversial, with contradictory evidence in the literature. We suggest that for accurate detection of *P*. *acnes*, it is critical to anticipate its existence in its biofilm form [5, 26, 27] and to use quantitative methods to determine bacteria counts. Unfortunately, this approach has not been utilized in any of the published studies in this field. In the current study, we homogenized disc samples to disrupt biofilm aggregates (which if undisrupted often lead to negative culture results [27]) to eliminate false negativity, and used quantitative anaerobic culture and real-time PCR to determine bacterial counts/genomes as one of the criteria to eliminate potential contamination and false positivity.

We prospectively enrolled 290 patients undergoing microdiscectomy for lumbar disc herniation, which is the largest series of patients to date, and examined their intraoperative disc biopsies by quantitative culture and real-time PCR. We isolated three bacterial species by culture: *P*. *acnes*, *CoNS* and *AHS* in 40%, 11% and 3% of cases, respectively. Our data are in agreement with a majority of the previous reports describing *P*. *acnes* and *CoNS* as the most frequently isolated by anaerobic culture [12, 14–17]. Overall the *P*. *acnes* positivity rate observed in our study is similar to the results of Stirling *et al*. [12], Arndt *et al*. [16] and Albert *et al*. [17], who observed 44%, 35% and 40% positivity rate, respectively. Analogically to our results, 5%, 14% and 31% prevalence of *CoNS* was described in three others studies [12, 14, 16].

We used standard surgical care protocols, including the use of prophylactic perioperative antibiotics (cefazolin), thus ensuring patient safety while conducting our research. We reasoned that cephalosporin prophylactic antibiotics would not affect the research results because these antibiotics do not penetrate into the intervertebral discs and because *P*. *acnes* biofilm within the disc is antibiotic resistant at the typically used doses [28]. In two protocols, prophylactic antibiotics were administered after collection of the biopsies to avoid inhibition of any bacteria in the sample [17], or were not administered at all [22]. In the first case, the positivity rate of *P*. *acnes* was identical with our study (40% in both), in the second study *P*. *acnes* positivity was only 4%, indicating that prophylactic cefazoline does not have any inhibitory effects on the *P*. *acnes* within the disc.

None of the previous studies determined *P*. *acnes* counts in the discs and distribution of the bacteria counts within their case series. We described the range of *P*. *acnes* counts obtained by culture and observed an 11% prevalence of intervertebral discs with abundant *P*. *acnes* (≥ 1x10^3^ CFU/ml). We suggest, that this quantity-adjusted prevalence is less biased by contamination than overall positivity. Similar prevalence of the *P*. *acnes* positive disc samples were observed by Agarwal *et al*. [15], Coscia *et al*. [14] and Zhou *et al*. [18] describing 13%, 19% and 24% positivity rate, respectively. As quantitative data from other studies are not available, and differences in the positivity rates differ significantly (from 4 to 44%), it is likely that some authors evaluated weak positivity as contamination and others as positivity.

However, there are also studies describing low or zero *P*. *acnes* positivity of discs in their case series [21–23]. For instance, *P*. *acnes* was not isolated from any of 30 disc samples in the study by Ben-Galim *et al*. [21], who implemented inoculation of cultures directly in the operating theatre to minimize the risk of contamination. Authors of this study suggest that the high prevalence of *P*. *acnes* observed in other studies mainly reflects contamination. We speculate that this unusual approach did not provide for sufficient homogenization of the tissue and disruption of *P*. *acnes* biofilms, which combined accounts for their negative culture results. Similarly, in the large-scale study on patients with chronic lower back pain, disc fragments were cultured directly without tissue processing or homogenization, and *P*. *acnes* were isolated in only 2 of 313 cases [23, 29].

We implemented a real-time PCR assay and laboratory contamination controls to accurately quantify *P*. *acnes* genomes directly in the disc tissue samples. The numbers of *P*. *acnes* genomes in the discs, which were *P*. *acnes* abundant by culture, were significantly higher when compared to non-abundant or *P*. *acnes* negative discs. There was no difference in the numbers of *P*. *acnes* genomes between the discs with non-abundant *P*. *acnes* and negative samples by culture, supporting a potential contamination origin of the disc samples with non-abundant *P*. *acnes* and accuracy of the empirical threshold (≥ 1x10^3^ CFU/ml) for the samples with abundant *P*. *acnes*. Only in 13 (4%) cases, were no *P*. *acnes* genomes detected. This is in agreement with outputs of large-scale studies based on next-generation sequencing of *P*. *acnes* DNA in a variety of clinical specimens concluding that when highly sensitive molecular methods are employed, *P*. *acnes* DNA could be detected in practically all sample types [30]. Moreover, it was shown that *P*. *acnes* DNA is a frequent contaminant of commercial Taq polymerases and PCR solutions [31], highlighting the necessity of quantitative information to distinguish between contamination and positivity [32]. Until now, only one team focused on direct detection of *P*. *acnes* DNA in disc tissue by qualitative end-point PCR [17]. Others used PCR only for verification of *P*. *acnes* DNA in purified colonies [18]. Albert *et al*. observed consistent results of culture and PCR methods; and, surprisingly, the cases negative by culture (60%) were negative by PCR [17], which could be explained by the decreased number of cycles (only 35) in their assay, probably to artificially decrease the sensitivity of their method.

We did not observe any significant association between *P*. *acnes* positivity and prevalence of previous spinal surgery and prior epidural steroid injection indicating endogenous origin of *P*. *acnes* and their role in degenerative disc disease more than their iatrogenic origin through disc contamination in the previous spinal surgeries. *P*. *acnes* positivity rates were independent of gender, type of herniation, and affected spinal segment. The average age of patients having discs with abundant *P*. *acnes* were lower (42 versus 48 years). We hypothesize, that *P*. *acnes* infection could accelerate regular mechanical, age-related, degenerative process in affected individuals and result in the necessity of earlier surgical intervention.

Also of note is the two most common commensals, *P*. *acnes* and coagulase-negative *staphylococci*, were also the two most common bacteria found in the disc tissue samples, which reflects the compelling need to gain an ability to discriminate between a contamination and an infection.

This study has several limitations. Aerobic cultures were not performed given disc tissue originates in a notably anaerobic environment. As we consider tissue homogenization a critical step of the experimental protocol, more standardized homogenization methods than mortar and pestle should be implemented. We assume that the biofilm form of *P*. *acnes* is typically present within the disc, but do not provide direct evidence of *P*. *acnes* biofilms from the disc tissue by microscopic methods. However, in the field of orthopedic infections, there is strong evidence of *P*. *acnes* forming a biofilm [26, 27]. As disc tissue from age-matched healthy individuals is not available, the identification of *P*. *acnes* in biopsies from comparable healthy individuals cannot be ruled out. Further, the lack of control cultures cannot exclude the possibility of contamination from the laboratory, operating room, surgical field, or skin. With exception of one [22], most of the previous studies that included negative controls proved that they are largely sterile [12, 14, 18]. Since positive endogenous controls do not necessarily connote contamination of the disc sample, we do not believe that endogenous controls are the optimal solution to identify contaminated disc samples. In our study, *P*. *acnes* counts and the number of *P*. *acnes* genomes were implemented as one of the criteria to eliminate potential contamination. The final answer with regard to distinguishing endogenous *P*. *acnes* infection of the disc from skin contamination will probably utilize comparative genomics to differentiate *P*. *acnes* genomes from the skin and disc tissue from a given individual [33, 34].

In conclusion, to our knowledge this is the first study that provides quantitative information about *P*. *acnes* in disc tissue samples in the largest series of patients with lumbar disc herniation–we demonstrated an 11% prevalence of the disc herniation samples with an abundance of *P*. *acnes*. Because “finding abundant microorganism in organisms suffering from the disease” is one of the most important of Koch’s postulates, in judging the relationship between a specific microbe and a specific disease, we believe that our findings support the theory that *P*. *acnes* is involved in the pathogenesis of at least a subset of patients with degenerative disc disease. Future studies are needed to evaluate relationship between *P*. *acnes* infection and clinical outcomes of these patients.
